# Capture and enrichment of CD34-positive haematopoietic stem and progenitor cells from blood circulation using P-selectin in an implantable device

**DOI:** 10.1111/j.1365-2141.2007.06967.x

**Published:** 2008-03

**Authors:** Joel C Wojciechowski, Srinivas D Narasipura, Nichola Charles, Deanne Mickelsen, Kuldeeepsinh Rana, Martha L Blair, Michael R King

**Affiliations:** 1 Department of Biomedical Engineering, University of RochesterRochester, NY, USA; 2 CellTraffix Inc.Rochester, NY, USA; 3 Department of Pharmacology and Physiology, University of RochesterRochester, NY, USA; 4 Department of Chemical Engineering, University of RochesterRochester, NY, USA

**Keywords:** P-selectin, haematopoietic progenitor, CD34, adult stem cell, implantable device

## Abstract

Clinical infusion of haematopoietic stem and progenitor cells (HSPCs) is vital for restoration of haematopoietic function in many cancer patients. Previously, we have demonstrated an ability to mimic physiological cell trafficking in order to capture CD34-positive (CD34^+^) HSPCs using monolayers of the cell adhesion protein P-selectin in flow chambers. The current study aimed to determine if HSPCs could be captured directly from circulating blood *in vivo*. Vascular shunt prototypes, coated internally with P-selectin, were inserted into the femoral artery of rats. Blood flow through the cell capture device resulted in a wall shear stress of 4–6 dynes/cm^2^. After 1-h blood perfusion, immunofluorescence microscopy and flow cytometric analysis revealed successful capture of mononuclear cells positive for the HSPC surface marker CD34. Purity of captured CD34^+^ cells showed sevenfold enrichment over levels found in whole blood, with an average purity of 28%. Robust cell capture and HSPC enrichment were also demonstrated in devices that were implanted in a closed-loop arterio-venous shunt conformation for 2 h. Adherent cells were viable in culture and able to differentiate into burst-forming units. This study demonstrated an ability to mimic the physiological arrest of HSPCs from blood in an implantable device and may represent a practical alternative for adult stem cell capture and enrichment.

Haematopoietic progenitors represent a small but important population of cells found in the blood circulation. These multipotent adult stem cells differentiate into every major blood cell type of the more than twenty trillion cells in the bloodstream. The lifespan of various blood cells can range from years for some lymphocytes, to months for erythrocytes, to mere hours for neutrophils, making continuous production of blood cells (haematopoiesis) necessary for even short term homeostasis (Gunsilius *et al*, 2001). Unfortunately, many of the most common cancer therapies are deleterious to haematopoietic function or even destroy it outright. Collection and enrichment of haematopoietic stem and progenitor cells (HSPCs) for infused restoration of haematopoiesis remains a vital means for cancer patient survival and a major area of research (To *et al*, 1997; Gunsilius *et al*, 2001).

Current methods of HSPC isolation focus on the immunological binding characteristics of antibodies to specific surface markers, such as CD34 or CD133, followed by separation using magnetic beads or cell sorting. These collection procedures result in a viable and specific population of HSPCs; however, the incubation process is time consuming and often results in contamination or inadequate cell yield. Less specific aphaeretic techniques attempt to increase the HSPC yield but result in an accompanying population of non-progenic cells that can be problematic for recipients due to either the reintroduction of malignant cells following autologous donation, or graft-versus-host disease (GVHD) following allogeneic donation (Horwitz & Sullivan, 2006). Furthermore, new studies suggest that immunologically separated HSPCs may lack an important stem cell phenotype. This phenotype represents a more primitive and less differentiated progenitor, which may be better suited for the reconstitution of haematopoietic function in the patient through improved engraftment rate and shortened time for engraftment (Engelhardt *et al*, 2002; Guo *et al*, 2003). Seemingly missing from the current landscape of this essential clinical procedure is a technique for the isolation of haematopoietic progenitors that lacks the negative aspects of immunological-based separation.

Recently, our laboratory has demonstrated that HSPCs exhibit a distinct behavioural phenotype when perfused over monolayers of P-selectin in microchannels (Charles *et al*, 2007). Discrete behaviour of rolling HSPCs includes decreased rolling velocity, decreased saltatory detachment, and increased number of adherent cells when compared with other blood cell types (such as leucocytes) flowing over similar monolayers. These observations support and extend previous results from another laboratory that showed similar behaviour by CD34-positive (CD34^+^) cells rolling on L-selectin substrates (Greenberg & Hammer, 2001). Selectin-mediated cell adhesion is a ubiquitous form of cell trafficking in the body. This mode of adhesion plays a major role in the arrest of cells from flow during vital physiological events, such as the tethering of neutrophils on the venular endothelium during the inflammatory response (Ley *et al*, 1995; Konstantopoulos *et al*, 2003; Kim & Sarelius, 2004), the capture of lymphocytes during the acquired immune defence (Springer, 1994), and the recently observed adhesion of leucocytes in arterioles (Sumagin & Sarelius, 2006). Furthermore, selectins are primary adhesion molecules for the homing of circulating HSPCs to the bone marrow during natural haematopoiesis (Schweitzer *et al*, 1996; Frenette *et al*, 1998; Peeled *et al*, 2000).

The aim of the current study was to determine if P-selectin could be used to capture and enrich a population of HSPCs from whole blood in the living animal. The inside lumenal surface of a blood-compatible plastic tube was coated with P-selectin and the tube was implanted into the femoral artery of an anesthetized rat to determine if CD34^+^ HSPCs could be captured from the circulation. Blood flow through implanted cell capture tubes coated with P-selectin enabled the collection of HSPCs with significant enrichment over the concentration found in whole blood. The captured HSPCs were viable and demonstrated a haematopoietic progenitor phenotype with an ability to differentiate into burst-forming units (BFU). We hypothesize that flow-mediated capture of HSPCs with P-selectin may represent a practical and more physiological alternative to HSPC isolation than the static immunological or density-mediated protocols currently used in the clinic.

## Materials and methods

### Preparation of implantable cell capture tubes

Under aseptic conditions, human recombinant P-selectin/Fc chimera (R&D Systems, Minneapolis, MN, USA) was adsorbed to the inside lumen of blood-compatible plastic tubing (inside diameter = 300 μm, length 50 cm, MicroRenathane MRE-025; Braintree Scientific Inc., Braintree, MA, USA) with a 2-h incubation of P-selectin solution (20 μg/ml or 40 μg/ml) diluted in Dulbecco's phosphate-buffered saline (PBS, GIBCO, Grand Island, NY, USA) at room temperature with one exchange of the solution midway through the incubation. Following a gentle PBS wash, non-specific blocking of the lumen surface was achieved with a 1-h incubation of either 5% sterile bovine serum albumin (BSA, Sigma-Aldrich, St. Louis, MO, USA) or 5% sterile milk protein (Nestle USA, Solon, OH, USA) dissolved in PBS. Following another PBS wash, the P-selectin layer was maintained in calcium-enriched Hanks’ balanced salt solution [2 mmol/l Ca^++^] (HBSS, GIBCO) until surgical implantation. Previous *in vitro* experiments have demonstrated that this adsorption procedure is effective for P-selectin mediated physiological rolling of cell populations (Charles *et al*, 2007). Similarly, separate tubes were prepared with a 2-h incubation of CD34 antibody (100 μg/ml, Santa Cruz Biotechnology, Santa Cruz, CA, USA) instead of P-selectin. Control tubes were prepared with a 2-h incubation of PBS only, followed by similar non-specific blocking.

### Surgical implantation of the device

Adult male Sprague-Dawley rats (Charles River Laboratories, Wilmington, MA, USA; body weight = 331 ± 6·2 g) were injected subcutaneously with granulocyte colony-stimulating factor (GCSF, Filgrastim, Amgen, Thousand Oaks, CA, USA) at 80 μg/kg body weight, daily for 3 d prior to surgery. In studies of human peripheral blood, the fraction of mononuclear cells (MNCs) positive for CD34 was typically less than 0·5% (Tong *et al*, 1994; de Wynter *et al*, 1998). GCSF-mobilization can increase this fraction to 1–2% or higher (Kim, 2003; Fietz *et al*, 2004; Nomura *et al*, 2004). Surgical procedures were performed under anaesthesia (pentobarbital sodium 26 mg/kg with chloral hydrate 128 mg/kg, intraperitoneal administration) using aseptic conditions as previously described (Blair & Mickelsen, 2006a,b). All experimental procedures were reviewed and approved by the University of Rochester's institutional animal care and use committee.

Two types of experiments were performed. In the first, animals were prepared for implantation of the cell-capture device via exposure of the right femoral vein and left femoral artery. A catheter was inserted into the right femoral vein for injection of supplemental anaesthetic. The left femoral artery was ligated distal to the point of tubing insertion. One end of the tubing was then inserted into the femoral artery through a small incision and threaded approximately 2 cm upstream towards the heart so that the tip of the tubing was in the aorta, 1–5 mm above the iliac bifurcation. A small bolus of the anticoagulant sodium heparin (150 μl, 1000 units/ml; Baxter Healthcare Co., Deerfield, IL, USA) was administered via the venous catheter, immediately after blood began to flow through the device tubing. Measurement of flow rate through the tube was accomplished via collection of outflow blood over timed intervals. Total blood flow through the tube was approximately 5 ml over a 1-h period, representing about 20% of the total blood volume of the rat (Blair & Mickelsen, 2006b). At the end of the 1-h perfusion period, the outflow end of the tubing was occluded and the cell capture device was withdrawn and prepared for analysis.

In the second set of experiments, the device was incorporated into the rat circulation via a closed-loop arterio-venous shunt, allowing blood that flowed through the device to recirculate. Animals utilized for these experiments were prepared identically to those used for the 1-h perfusion experiments, except that the outflow end of the tube was inserted into the left femoral vein and advanced approximately 2 cm into the vena cava. In this way, blood entered the tubing from the arterial circulation and then returned to the animal's circulation from the venous end of the tubing. Following a 2-h period of blood flow through the cell capture device, the venous end of the tubing was occluded and the device was removed for analysis.

All animals were maintained in an anesthetized state throughout the procedure and were euthanized with an overdose of anaesthesia.

### Collection and analysis of captured cells

Following removal from the rat's circulation, cell capture tubes were positioned on an inverted IX-81 epi-fluorescence microscope (Olympus USA, New York, NY, USA) coupled to an intensified CCD camera (SensiCam QE, Cooke PCO, Auburn Hills, MI, USA) and brightfield CCD camera (Hitachi Kokusai Electronic Inc., Tokyo, Japan) for direct visualization of the adherent cells in the tube lumen. Tubes were rinsed of residual blood and non-adherent cells with a gentle wash of HBSS [2 mmol/l Ca^++^] for 20 min (0·5 dynes/cm^2^). Twenty representative fields were imaged to determine adherent cell concentration in the capture tube. Adherent cells were then further analysed either by direct fluorescent visualization or by flow cytometry, or were utilized in cell culture experiments to characterize expansion as described in the following section.

For capture tubes evaluated by direct visualization, the tube lumen was incubated for 30 min with a monoclonal antibody against the haematopoietic stem cell surface marker CD34 (Santa Cruz Biotechnology) conjugated to quantum dots (Qdot 605, Molecular Probes, Eugene, OR, USA). Following a gentle rinse, representative fields were imaged and analysed for CD34-positive cells determined by increased fluorescence intensity relative to CD34-negative leucocytes.

For capture tubes evaluated with quantitative flow cytometry analysis of CD34-positive cells, adherent cells were extracted from the tubes using increased shear rate and air embolism. Cells were then split into equal samples and incubated for 30 min with either CD34 antibody (Santa Cruz Biotechnology) or matched isotype control antibody (Santa Cruz Biotechnology). Following wash and resuspension, cell fractions were run on the FACSCalibur system (Becton-Dickinson, San Jose, CA, USA). Cytometric data was analysed using FACSCalibur software on a Macintosh G3 computer (Apple Inc., Cupertino, CA, USA). Quantification of the CD34-positive cell population of peripheral blood in GCSF-mobilized and non-mobilized rats was determined through flow cytometric analysis of erythrocyte-lysed blood from the same animals in which cell capture tubes were implanted.

### Expansion of captured haematopoietic stem cells

In experiments performed on a separate group of implanted rats, adherent blood-borne cells were extracted from P-selectin capture shunts (40 μg/ml) and screened for viable haematopoietic progenitors using a colony-forming cell (CFC) assay (HSC005, R&D Systems). Briefly, following positive trypan blue staining of a separate fraction, 10^5^ cells in 100 μl were mixed with 1 ml methylcellulose enriched media. Cells were plated in six-well plates and incubated at 37°C, 5% CO_2_ for up to 28 d. For a comparison of same-species haematopoietic progenitor expansion, MNCs were extracted from rat whole blood via Ficoll gradient separation and plated in the same manner (separate platings of 10^5^ and 10^6^ cells). Following 14 d, BFU and colony-forming units (CFU) were observed, counted, and imaged under the microscope for up to 28 d.

### Statistics

Statistical analysis of MNC capture density and CD34^+^ cell purity for 1-h perfusion experiments was performed by analysis of variance (anova), followed by Holm-Sidak *post hoc* comparisons between individual experimental groups (SPSS SigmaStat 3.0 software, SPSS, Inc., Chicago, IL, USA) using an overall significance level of *P* < 0·05. All other data were analysed by Student *t*-test (Prism 3.0; GraphPad Software, San Diego, CA, USA) at a level of significance *P* < 0·05. Data are presented as the mean ± SE.

## Results

### MNC adhesion to implanted capture tubes

**Fig 1:**
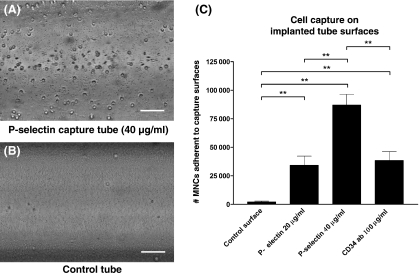
Direct capture of blood-borne nucleated cells from circulation using P-selectin and non-coated control surfaces in implanted devices. Following incorporation into the femoral artery of anesthetized rats and 1-h blood perfusion, P-selectin coated tubes (A) showed a significantly greater average concentration of captured nucleated cells than non-coated control tubes (B) [184·6 ± 19·9 cells/mm^2^ for P-selectin tubes (40 μg/ml) vs. 4·7 ± 1·4 cells/mm^2^ for control surfaces (*P* < 0·01), bar = 50 μm]. (C) Total cell yields from 50 cm implanted tubes with cell adhesion molecule surfaces were significantly greater than the yield from non-specific binding in control tubes (**P < 0·01).

To facilitate cell capture on the implanted device surface using physiological flow-mediated mechanisms, tubes with lumenal surfaces adsorbed with P-selectin were incorporated into blood circulation via insertion into the femoral artery of anesthetized rats. This permitted blood to be redirected from the rat's circulation into the capture device, with flow through the tubing driven by the rat's arterial blood pressure. To optimize the shear stress environment at the capture surface for cell adhesion, a tube length of 50 cm was used to produce the appropriate volumetric flow given fluid resistance and blood pressure. The average flow rate through the implanted tubes was 81·5 ± 2·2 μl/min (mean ± SE, *n* = 47 rats), corresponding to a wall shear stress of approximately 5 dynes/cm^2^. Following extraction and gentle washing of residual erythrocytes and non-adherent cells, the number of adherent MNCs (including polymorphonuclear or PMN cells) was quantified by direct microscopy. Lumenal surfaces of tubes coated with P-selectin or antibody against CD34 showed robust and sustained adhesion of MNCs when compared to non-coated control tubes. Figure 1 demonstrates the ability of adhesion molecule-coated tubes to capture blood-borne nucleated cells over a 1-h period during which blood flowed directly from the rat's circulatory system through the capture tube. Tubes that had been incubated with 20 μg/ml or 40 μg/ml P-selectin captured an average of over 34 000 and 87 000 nucleated cells, respectively, corresponding to surface concentrations of 72·7 ± 16·9 cells/mm^2^ (*n* = 5) and 184·6 ± 19·9 cells/mm^2^ (*n* = 6). Tubes with capture layers comprised of adhesion molecules all showed significantly increased MNC adhesion when compared to non-coated control tubes, which showed little cellular arrest (4·7 ± 1·4 cells/mm^2^, *n* = 6). Similar concentrations of platelets were adherent in both P-selectin coated tubes and in control tubes (as observed in Fig 1A and B).

Successful adsorption of P-selectin to the tubes during incubation was verified with a protein deposition assay that established a minimum of 55% of the soluble P-selectin chimera being taken up by the capture surface using 40 μg/ml P-selectin solution (data not shown). A P-selectin solution of 20 μg/ml resulted in deposition of at least 65% of the adhesion molecule. Overall cell adhesion was similar between upstream and downstream ends of the capture tubes, supporting existing evidence that mechanisms of adhesion during blood cell recruitment take place on the micrometre length scale (Kunkel *et al*, 2000; King *et al*, 2003; Kim & Sarelius, 2004).

### Capture of CD34-positive haematopoietic stem cells

**Fig 2:**
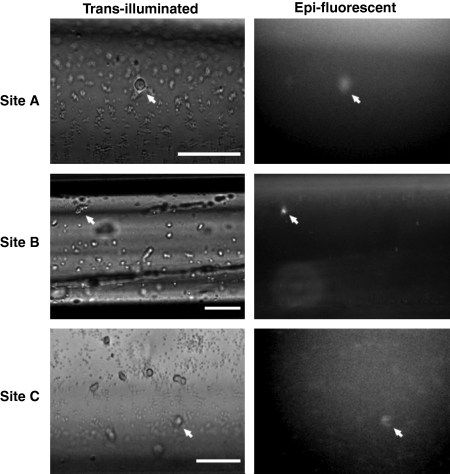
Paired brightfield and epi-fluorescence images of captured cells immunostained for CD34. Comparison of representative paired brightfield and fluorescent images of adherent cells immunolabelled with an antibody against a hematopoietic stem cell surface marker conjugated to quantum dots revealed blood-borne CD34-bright cells (white arrows) distinctly visible among CD34-dark cells on the capture surface (bar = 50 μm). Quantitative analysis confirmed a sixfold enrichment in the purity of CD34-positive cells on P-selectin surfaces over the proportion found in rat whole blood.

To determine if P-selectin coated tubes successfully captured blood-borne HSPCs, the captured cell population was labelled with an antibody against the HSPC surface marker CD34 conjugated to fluorescent quantum dots. We observed paired images of the tube's lumenal surface under trans-brightfield and epi-fluorescence illumination. Despite a small amount of unavoidable autofluorescence from the tube material itself, bright CD34-positive cells were clearly evident among nucleated CD34-negative cells captured from the bloodstream (Fig 2).

**Fig 3:**
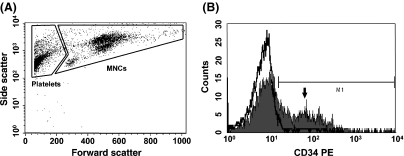
Flow-cytometric analysis of the adherent blood-borne cells from implanted tubes indicated a distinct population of CD34-positive cells on the capture surface. (A) Following extraction from the cell-capture tube, adherent mononuclear cells (MNCs) were analysed for surface expression of CD34 (the left-hand region of smaller platelets was excluded from analysis). (B) Immunofluorescent flow cytometric analysis of captured cells revealed a distinct population of MNCs positive for CD34 (arrow, subpopulation represents 38% of total MNCs) when compared to cells stained with isotype control antibody (bold line).

**Fig 4:**
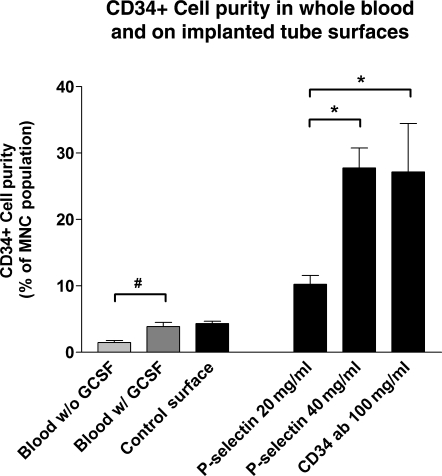
Purity of CD34-positive cells on implanted cell-capture devices with various adhesion-molecule surfaces and in whole blood of granulocyte colony-stimulating factor (GCSF)-mobilized and non-mobilized rats. As expected, the presence of CD34-positive cells was significantly higher in whole blood of GCSF-mobilized rats *versus* their non-mobilized counterparts (#*P* < 0·05). Following incorporation into the femoral artery of anesthetized rats and 60-min blood perfusion, purity of CD34-positive cells among mononuclear cells captured using P-selectin was elevated sevenfold over that of whole blood and non-coated control surfaces. CD34^+^ cell-purity in tubes incubated with 40 μg/ml P-selectin was significantly greater than that from capture surfaces coated with less P-selectin, and was similar to surfaces coated with CD34 antibody (*P < 0·05).

In experiments conducted to fully quantify HSPC capture using flow cytometry, captured cell populations were extracted from tubes and stained with a fluorescent antibody against CD34 (paired samples stained with matched isotype antibody were analysed as a control). Figure 3A illustrates representative flow cytometric forward- and side-scatter data of the extracted cells from an implanted tube coated with P-selectin, with gates established to discern captured MNCs from debris and smaller adherent platelets. Immunofluorescence flow cytometric analysis revealed that, when compared to matched-isotype control antibody, MNCs adherent to capture surfaces included a distinct population of CD34^+^ cells (Fig 3b). Quantification of the ability of implanted tubes to capture CD34^+^ cells was determined by measuring CD34^+^ cell purity, defined as the percentage of captured CD34^+^ cells to total captured MNCs. Figure 4 illustrates the average level of CD34^+^ cell purity from each implantable cell capture tube type compared with that of circulating blood in rats. Purity of CD34^+^ cells on non-coated control tubes was similar to that found in blood circulation [4·3 ± 0·3% vs. 3·9 ± 0·6% for control tubes (*n* = 3 rats) and whole rat blood (*n* = 5), respectively]. Conversely, capture tubes coated with cell adhesion molecules had robust cell adhesion and significantly higher CD34^+^ cell purity. For example, tubes incubated with 20 μg/ml and 40 μg/ml P-selectin resulted in an average purities of 10·3 ± 1·3% (*n* = 4) and 27·8 ± 3·0% (*n* = 6), respectively, the latter reflecting a sevenfold enrichment of CD34^+^ cells over whole blood (Fig 4). Tubes incubated with antibody to CD34 (100 μg/ml) elicited a CD34^+^ cell purity that was similar to P-selectin tubes (27·2 ± 7·3%, *n* = 4). However, as shown in Fig 1, CD34 antibody tubes exhibited inferior flow-mediated cell capture when compared to P-selectin tubes (40 μg/ml).

The similarity in purities between non-coated control tubes and whole blood establishes two important points when considering cellular capture and enrichment within P-selectin tubes. First, non-coated control tubes showed dramatically less cellular adhesion than tubes designed for active capture (by nearly two orders of magnitude), indicating that non-specific cellular adhesion is inconsequential and does not contribute to the CD34^+^ cell enrichment on active capture surfaces. Second, the fact that non-specific binding, though minimal, results in CD34^+^ purity similar to that in whole blood, suggests that CD34^+^ enrichment in P-selectin tubes is the result of the specific molecular interactions governing cellular adhesion.

### Capture of haematopoietic stem cells in fully implanted arterio-venous devices

**Fig 5:**
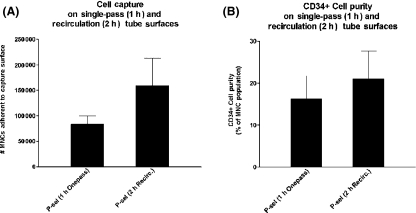
Mononuclear cell (MNC) capture and CD34-positive cell purity from implanted cell-capture devices inserted into the rat femoral artery and perfused with a single-pass of blood for 1 h, or incorporated completely into the circulatory system of the rat via arterial-venous shunt for continuous 2-h recirculation. (A) The average capture of MNCs in 50 cm P-selectin tubes (40 μg/ml) was 84 036 cells (178·3 ± 33·8 cells/mm^2^, *n* = 6) for 1-h single blood-pass and 159 264 cells (338·0 ± 115·0 cells/mm^2^, *n* = 7) for 2-h recirculation. (B) Purity of captured MNC populations for CD34^+^ cells was 16·2 ± 5·5% and 21·0 ± 6·6% for 1- and 2-h experiments, respectively. The results confirm that targeted cell capture also occurs in a fully implanted arterio-venous recirculation device, and suggest that the amount of capture and degree of purity might be increased with longer times of perfusion.

Finally, in separate experiments, cell capture tubes were completely incorporated into the circulatory system of the rat via arterio-venous shunt, allowing blood that entered the tubing from the arterial circulation to be returned to the animal's circulation from the downstream (venous) end of the tubing over a 2-h time period of continuous perfusion. MNC capture and CD34^+^ cell purity were compared with that from tubes inserted into the femoral artery of the rat and perfused, as before, for 1 h with a single pass of blood. Figure 5 illustrates that, similar to previous experiments, the capture of MNCs in 50-cm P-selectin tubes (40 mg/ml) with a 1-h single blood-pass was maintained at over 84 000 cells, or 178·3 ± 33·8 cells/mm^2^ (*n* = 6). Two-hour perfusion through the arterio-venous recirculation shunts resulted in the arrest of nearly 160 000 MNCs, or 338·0 ± 115·0 cells/mm^2^ (*n* = 7; *P* > 0·05). Purity of captured populations for CD34^+^ cells were 16·2 ± 5·5% and 21·0 ± 6·6% for 1- and 2-h experiments, respectively. These results confirm that targeted cell capture also occurs in a fully implanted arterio-venous recirculation device, and suggest that the amount of capture and degree of purity might be increased with longer times of perfusion.

### Viability and expansion of haematopoietic stem cells

**Fig 6:**
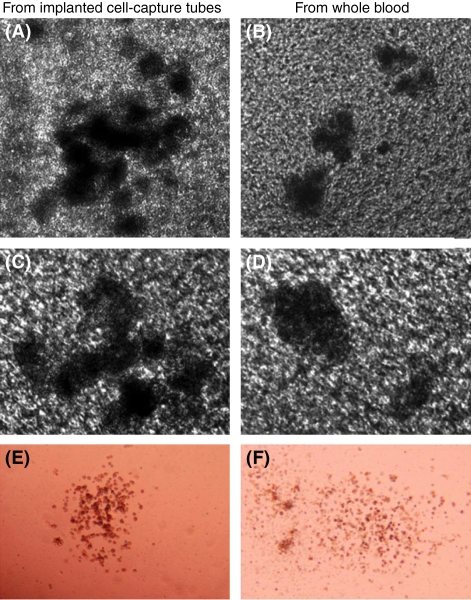
Bright field microscopic images of erythroid burst-forming units and granulocyte/monocyte colony forming units, expanded from hematopoietic stem cells (HSCs) harvested from implanted cell capture tubes and whole blood. Viable blood-borne HSCs from implanted P-selectin coated tubes (A, C, and E) and rat whole blood (B, D, and F) were expanded in culture for up to 28 d. Robust colonies were observed from HSCs in each group.

The enriched population of blood-borne cells on the device surface was screened to verify the ability to capture and maintain viable progenitors with haematopoietic colony-forming potential. Cells extracted from implanted P-selectin tubes (40 μg/ml) showed over 90% viability and were expanded in culture using a CFC assay. Six-well plates were seeded at a concentration of 10^5^ P-selectin tube-captured cells, as well as concentrations of 10^5^ and 10^6^ MNCs isolated from rat peripheral blood. Figure 6 compares representative images from 3 to 4-week-old cultures of cells obtained from P-selectin capture tubes (Fig 6A, C, and E) with MNCs from whole rat blood known to contain populations of healthy HSPCs (Fig 6B, D, and F). In both groups, hemoglobinized erythroblasts and granulocyte/monocyte precursors were observed in both BFU and CFU (BFU-E and CFU-GM).

Table I demonstrates the haematopoietic expansion of HSPCs, given as the number of BFU-E and CFU-GM colonies after 3–4 weeks. HSPCs from P-selectin tubes were observed to expand similarly to HSPCs from whole blood when plated at similar concentrations. It is interesting to note that, while the plating of 10^5^ cells for expansion (dictated by the cell yield of our device) and the use of a cross-species colony assay (due to unavailability of rat-specific CFC media) were seemingly limiting factors to colony expansion, the presence of robust erythroid and granulocyte/monocyte colonies indicates that implantable P-selectin tubes have the capacity not only for HSPC capture and enrichment, but also for short-term maintenance of viable progenitors with the potential for differentiation and proliferation.

**Table I:** Hematopoietic expansion potential of hematopoietic stem and progenitor cells (HSPCs) from P-selectin capture tubes and peripheral blood, given as the number of hemoglobinized erythroblasts and granulocyte/monocyte precursors were observed in both BFU and CFU colonies after 3–4 weeks.

Hematopoietic Colony Type	P-selectin Captured MNCs (10^5^ cells)	Peripheral Blood MNCs (10^5^ cells)	Peripheral Blood MNCs (10^6^ cells)
CFU-GM (# colonies)	1.5 (2 of 5 plates active)	1.5 (2 of 7 plates active)	14.4 (5 of 6 plates active)
BFU-E (# colonies)	1.5 (2 of 5 plates active)	0.5 (2 of 7 plates active)	4.0 (5 of 6 plates active)

Viable blood-borne HSPCs from implanted P-selectin coated tubes and rat whole blood expand to similar numbers of colony-types in culture (counts are given as average number of colonies per active plate). The presence of erythroid and granulocyte/monocyte colonies indicates that implantable P-selectin tubes have the capacity not only for HSPC capture and enrichment, but also for short-term maintenance of viable progenitors with the potential for differentiation and proliferation.

## Discussion

We have demonstrated an ability to capture and enrich haematopoietic stem cells from the bloodstream of a living animal using P-selectin in an implanted device. To our knowledge, the current study represents the first time that P-selectin has been used to sequester adult stem cells directly from circulation *in vivo*, rather than through immunological or density gradient processing of blood samples. This flow-based system mimics physiological events involved in normal cellular trafficking, such as the recruitment of leucocytes during the inflammatory response or homing of HSPCs to the bone marrow. The enriched population of captured HSPCs showed significantly increased purity over the population found in whole blood or on control surfaces. Moreover, extracted cells were viable and could be expanded in culture to produce BFU and CFU. This approach represents a possible alternative to the current HSPC enrichment protocols that are widely utilized in the clinic as an adjunct to specific cancer therapies.

Findings from the current study support and extend previous work from our and other laboratories demonstrating capture of CD34^+^ cells on selectin surfaces in isolated systems (Greenberg & Hammer, 2001). We speculate that significant enrichment of CD34^+^ cells from the bloodstream in an implantable device, especially in an environment filled with abundant binding competitors, such as leucocytes, is facilitated by differences in affinity and adhesion kinetics between cell types in the heterogeneous population of blood. Earlier work in our laboratory has shown that populations of CD34^+^ cells exhibit a rolling phenotype that is quite different from that of leucocytes or other CD34− cells when interacting with selectins in flow microchannels (Charles *et al*, 2007). In those studies CD34^+^ cells were observed to have lower rolling velocities, decreased saltatory behaviour, and lower detachment rates than leucocytes perfused over the same surface. Additional studies have shown that within venular microvessels *in situ*, leucocyte attachment is quite transient and short-lived (Wojciechowski & Sarelius, 2005; Ryschich *et al*, 2006). Under unstimulated or lightly-activated conditions, up to 90% of adherent leucocytes are known to detach into the bulk flow within 15 min of initial capture (Wojciechowski & Sarelius, 2005). Thus, while capture of a large and varied population of blood-borne cells may occur initially, over time the different binding kinetics of the various cell types are likely to result in a more enriched population of adherent cells, such as HSPCs. In other words, we surmise that the enrichment of CD34^+^ cells on the capture layer is a consequence of a temporally evolved system in which leucocytes or other MNCs are displaced over time by HSPCs having greater adhesion and lower detachment rates. The potential clinical applicability of this device is further supported by results from the current study demonstrating robust MNC capture and enriched CD34^+^ cell purity in capture tubes implanted as an arterio-venous shunt through which blood could be returned to circulation. Longer-term temporal dependence of HSPC binding will be examined further in future studies where capture tubes will be incorporated into blood circulation on the order of days.

Despite significant enrichment of HSPCs from circulating blood in the current study, it is likely that modification and optimization of the system would result in further increased target cell purity and yield. As an example, while the length of our system was chosen to facilitate flow rates and shear stresses amenable to selectin-mediated cell capture (on the order of 5 dynes/cm^2^), the cross-section of the current system was matched to the anatomical diameter of the femoral artery of the rat. A decrease in the diameter of the flow tubes to less than 100 μm, with a length-width geometry adjusted for optimal shear stress, should result in hemodynamic effects more favourable for blood-borne cell capture, such as enhanced margination. This, in turn, would enable a larger survey of nucleated cells in bulk flow and a potential increase in the capture of stem cells.

One measure of potential efficacy of our system is demonstrated by comparing the CD34^+^ cell purity achieved using P-selectin with that of cell-capture surfaces comprised of an antibody against a stem cell surface marker directly. In this manner, the average purity achieved with our P-selectin tubes in a non-optimized geometry matched that achieved using antibody against CD34. It is possible that the achievable purity of CD34^+^ cells in optimized selectin capture devices could surpass those of antibody capture devices as a result of the preferred flow-mediated nature of selectin binding *versus* that of antibodies, whose use in a flow-mediated system is less than optimal because of the longer time required for bond formation.

The use of P-selectin for flow-mediated enrichment of haematopoietic progenitors holds the potential not only to improve on current cell-isolation techniques in basic science and laboratory animal research (as we have shown), but holds a potential for translation to human benefit in the clinic. Results from recent studies have shown that positive screening for haematopoietic progenitors can reduce the tumour cell load during autologous donation and may play a role in minimizing infusion-related toxicity (Shpall *et al*, 1997; Mohr *et al*, 1999; Kasow *et al*, 2007). Additionally, there is compelling evidence to suggest that purification of CD34^+^ progenitors during allogeneic donation effectively minimizes GVHD and may reduce relapse incidence (Lang *et al*, 2003; Lee *et al*, 2005). Thus, the use of a flow-mediated system for haematopoietic progenitor enrichment maintains the potential to be applied across autologous and allogeneic donor origins. Moreover, because our cell-capture system mimics the same selectin-mediated trafficking used by the body to induce engraftment of haematopoietic progenitors in the bone marrow, we hypothesize that its use may not only carry with it the demonstrated benefits of haematopoietic progenitor enrichment, but may elicit an improved clinical response through the capture of a superior population of stem cell – a population possibly overlooked by immunological separation techniques. We reason that the ideal method to capture cells intended for engraftment to the haematopoietic niche, is to simulate the very conditions of selectin-mediated trafficking within the target tissue *in situ*.

In summary, our study demonstrates an ability to directly capture and enrich a small but important population of blood-borne cells from circulation using P-selectin in an implantable device. Selective capture of HSPCs resulted in enrichment of up to sevenfold over the population found in whole blood. Extracted cells were viable and able to be expanded in culture to promote differentiated haematopoietic colonies. These results not only represent a potential alternative approach to stem cell enrichment for both research and clinical purposes, but also suggest a method to target other rare populations of circulating cells such as cancer cells *in vivo*.

## References

[b1] Blair ML, Mickelsen D (2006a). Activation of lateral parabrachial nucleus neurons restores blood pressure and sympathetic vasomotor drive after hypotensive hemorrhage. American Journal of Physiology. Regulatory, Integrative and Comparative Physiology.

[b2] Blair ML, Mickelsen D (2006b). Plasma protein and blood volume restitution after hemorrhage in conscious pregnant and ovarian steroid-replaced rats. American Journal of Physiology. Regulatory, Integrative and Comparative Physiology.

[b3] Charles N, Liesveld JL, King MR (2007). Investigating the feasibility of stem cell enrichment mediated by immobilized selectins. Biotechnology Progress.

[b4] Engelhardt M, Lubbert M, Guo Y (2002). CD34(+) or CD34(−): which is the more primitive?. Leukemia.

[b5] Fietz T, Rieger K, Dimeo F, Blau IW, Thiel E, Knauf WU (2004). Stem cell mobilization in multiple myeloma patients: do we need an age-adjusted regimen for the elderly?. Journal of Clinical Apheresis.

[b6] Frenette PS, Subbarao S, Mazo IB, von Andrian UH, Wagner DD (1998). Endothelial selectins and vascular cell adhesion molecule-1 promote hematopoietic progenitor homing to bone marrow. Proceedings of the National Academy of Sciences of the United States of America.

[b7] Greenberg AW, Hammer DA (2001). Cell separation mediated by differential rolling adhesion. Biotechnology and Bioengineering.

[b8] Gunsilius E, Gastl G, Petzer AL (2001). Hematopoietic stem cells. Biomedicine and Pharmacotherapy.

[b9] Guo Y, Lubbert M, Engelhardt M (2003). CD34− hematopoietic stem cells: current concepts and controversies. Stem Cells.

[b10] Horwitz ME, Sullivan KM (2006). Chronic graft-versus-host disease. Blood Reviews.

[b11] Kasow KA, Sims-Poston L, Eldridge P, Hale GA (2007). CD34(+) hematopoietic progenitor cell selection of bone marrow grafts for autologous transplantation in pediatric patients. Biology of Blood and Marrow Transplantation.

[b12] Kim YH (2003). Intramyocardial transplantation of circulating CD34+ cells: source of stem cells for myocardial regeneration. Journal of Korean Medical Science.

[b13] Kim MB, Sarelius IH (2004). Role of shear forces and adhesion molecule distribution on P-selectin-mediated leukocyte rolling in postcapillary venules. American Journal of Physiology. Heart and Circulatory Physiology.

[b14] King MR, Kim MB, Sarelius IH, Hammer DA (2003). Hydrodynamic interactions between rolling leukocytes in vivo. Microcirculation.

[b15] Konstantopoulos K, Hanley WD, Wirtz D (2003). Receptor-ligand binding: ‘catch’ bonds finally caught. Current Biology.

[b16] Kunkel EJ, Dunne JL, Ley K (2000). Leukocyte arrest during cytokine-dependent inflammation in vivo. Journal of Immunology.

[b17] Lang P, Handgretinger R, Niethammer D, Schlegel PG, Schumm M, Greil J, Bader P, Engel C, Scheel-Walter H, Eyrich M, Klingebiel T (2003). Transplantation of highly purified CD34+ progenitor cells from unrelated donors in pediatric leukemia. Blood.

[b18] Lee SH, Lee MH, Lee JH, Min YH, Lee KH, Cheong JW, Lee J, Park KW, Kang JH, Kim K, Kim WS, Jung CW, Choi SJ, Park K (2005). Infused CD34+ cell dose predicts long-term survival in acute myelogenous leukemia patients who received allogeneic bone marrow transplantation from matched sibling donors in first complete remission. Biology of Blood and Marrow Transplantation.

[b19] Ley K, Bullard DC, Arbones ML, Bosse R, Vestweber D, Tedder TF, Beaudet AL (1995). Sequential contribution of L- and P-selectin to leukocyte rolling in vivo. Journal of Experimental Medicine.

[b20] Mohr M, Hilgenfeld E, Fietz T, Hoppe B, Koenigsmann M, Hoffmann M, Knauf WU, Cassens U, Sibrowski W, Kienast J, Thiel E, Berdel WE (1999). Efficacy and safety of simultaneous immunomagnetic CD34+ cell selection and breast cancer cell purging in peripheral blood progenitor cell samples used for hematopoietic rescue after high-dose therapy. Clinical Cancer Research.

[b21] Nomura S, Inami N, Kanazawa S, Iwasaka T, Fukuhara S (2004). Elevation of platelet activation markers and chemokines during peripheral blood stem cell harvest with G-CSF. Stem Cells.

[b22] Peled A, Kollet O, Ponomaryov T, Petit I, Franitza S, Grabovsky V, Slav MM, Nagler A, Lider O, Alon R, Zipori D, Lapidot T (2000). The chemokine SDF-1 activates the integrins LFA-1, VLA-4, and VLA-5 on immature human CD34(+) cells: role in transendothelial/stromal migration and engraftment of NOD/SCID mice. Blood.

[b23] Ryschich E, Kerkadze V, Lizdenis P, Paskauskas S, Knaebel HP, Gross W, Gebhard MM, Buchler MW, Schmidt J (2006). Active leukocyte crawling in microvessels assessed by digital time-lapse intravital microscopy. Journal of Surgical Research.

[b24] Schweitzer KM, Drager AM, van der Valk P, Thijsen SF, Zevenbergen A, Theijsmeijer AP, van der Schoot CE, Langenhuijsen MM (1996). Constitutive expression of E-selectin and vascular cell adhesion molecule-1 on endothelial cells of hematopoietic tissues. American Journal of Pathology.

[b25] Shpall EJ, LeMaistre CF, Holland K, Ball E, Jones RB, Saral R, Jacobs C, Heimfeld S, Berenson R, Champlin R (1997). A prospective randomized trial of buffy coat versus CD34-selected autologous bone marrow support in high-risk breast cancer patients receiving high-dose chemotherapy. Blood.

[b26] Springer TA (1994). Traffic signals for lymphocyte recirculation and leukocyte emigration: the multistep paradigm. Cell.

[b27] Sumagin R, Sarelius IH (2006). TNF-alpha activation of arterioles and venules alters distribution and levels of ICAM-1 and affects leukocyte-endothelial cell interactions. American Journal of Physiology. Heart and Circulatory Physiology.

[b28] To LB, Haylock DN, Simmons PJ, Juttner CA (1997). The biology and clinical uses of blood stem cells. Blood.

[b29] Tong J, Hoffman R, Siena S, Srour EF, Bregni M, Gianni AM (1994). Characterization and quantitation of primitive hematopoietic progenitor cells present in peripheral blood autografts. Experimental Hematology.

[b30] Wojciechowski JC, Sarelius IH (2005). Preferential binding of leukocytes to the endothelial junction region in venules in situ. Microcirculation.

[b31] de Wynter EA, Buck D, Hart C, Heywood R, Coutinho LH, Clayton A, Rafferty JA, Burt D, Guenechea G, Bueren JA, Gagen D, Fairbairn LJ, Lord BI, Testa NG (1998). CD34+AC133+ cells isolated from cord blood are highly enriched in long-term culture-initiating cells, NOD/SCID-repopulating cells and dendritic cell progenitors. Stem Cells.

